# Central and Peripheral Retina Arise through Distinct Developmental Paths

**DOI:** 10.1371/journal.pone.0061422

**Published:** 2013-04-16

**Authors:** Sara J. Venters, Takashi Mikawa, Jeanette Hyer

**Affiliations:** 1 Department of Neurosurgery, University of California San Francisco, San Francisco, California, United States of America; 2 Department of Ophthalmology, University of California San Francisco, San Francisco, California, United States of America; 3 Cardiovascular Research Institute, University of California San Francisco, San Francisco, California, United States of America; University of Dayton, United States of America

## Abstract

In the mature eye, three distinct tissue fates, retina, ciliary body, and iris, arrange with a strict linear organization along the central (back) to peripheral (front) axis. The establishment of this topographical relationship within the optic vesicle is not well understood. We use a targeted vital labeling strategy to test the derivation of mature eye tissues from the optic vesicle of the chick embryo. Fate mapping uncovers two distinct origins of the neural retina. Contrary to expectations, the central neural retina has a discrete origin within the posterior optic vesicle. The peripheral retina derives from the distal optic vesicle, sharing a common origin with more peripheral tissue fates. This study identifies for the first time two distinct retinal sub-domains, central and peripheral, which arise during embryogenesis. Identification of these discrete retinal compartments provides a framework for understanding functional and disease processes throughout retinal tissue.

## Introduction

The morphogenesis of the vertebrate eye begins as a lateral evagination of the anterior neural tube. As development proceeds, continued lateral expansion creates a distinct optic vesicle that is in contact with the surface ectoderm. During the growth process in the chick, the simple vesicle contorts and shifts, as neural crest and paraxial mesoderm invade the area; this can be observed in transverse sections through the region [1], [2]. After a period of contact with the overlying surface ectoderm, a portion of the distal optic vesicle (OV) invaginates. Invagination creates a bilayered optic cup (OC) with an inner and outer layer, and a lip or hingepoint where the epithelium folds back upon itself. The invagination is most apparent in the dorsal aspect of the vesicle; the ventral half of the eye does not fully invaginate until after an additional day of development [2]. Once the optic cup forms, the inner layer differentiates as the sensory neural retina, and the outer layer differentiates as retinal pigmented epithelium (RPE). The optic cup lip (OCL), which is formed as a result of optic cup morphogenesis, acts a progenitor pool for further peripheral eye growth. The OCL becomes the pupil margin in the fully differentiated eye [3].

The mature optic cup is patterned along all three of its axes (dorsal-ventral (DV), nasal-temporal (NT), and central-to-peripheral [also commonly referred to as anterior-posterior, with anterior being closest to the lens, as in the anterior segment]). Dorsal-Ventral and Nasal-Temporal patterning influence how the retinal neurons connect to the brain. Central to peripheral pattering in the OC results in distinct tissues (retina, ciliary body, iris), occupying progressively central to peripheral positions within the eye. During development, this axis is observed in the central to peripheral gradient of neuronal maturation in the retina and the difference in neuronal derivatives from central versus peripheral progenitors [4], [5], [6], [7], [8], [9], [10].

A lineage boundary has been described along the dorsal-ventral axis of the eye, and is most likely established during optic vesicle stages [11]. However, there is no assessment of a lineage boundary along the central-to-peripheral axis. In a previous study, we demonstrated that much of the mature eye was derived from cells that originally resided at the optic cup lip, including significant portions of the peripheral retina. OCL-derived cells did not populate the central retina [3]. Peripheral retina was not, therefore, an expansion of the central retinal domain, but part of the ongoing central to peripheral growth and maturation gradient of the optic cup from the optic cup lip.

There are several unique anatomical structures that define the central retina in adult eyes and yet very little understanding of how these features develop. Although there are inter- and intra-species differences (for example, birds have an avascular retina, and among mammals, only primates have a fovea centralis), all vertebrates have their area of highest visual acuity in the central retina. This region is often cone-rich, a zone where the neuroblasts have never produced rod photoreceptors. There are human syndromes that preferentially affect the development of central retina/macula, while the peripheral retina develops normally [12]. A central-to-peripheral axis has been described by fish mutants, which identify genetic differences between central and peripheral eye development [13], [14]. Together with our previous lineage tracing, these studies are consistent with potential functional differences in central versus peripheral retina arising as a result of underlying differences in developmental histories.

The present study shows that domains giving rise to A) central neural retina, B) central RPE, or C) optic cup lip can be identified in the OV. Contrary to expectations, the distal tip of the optic vesicle gives rise to the OCL and not the central neural retina within the optic cup. Our findings lead to a new fate map of the OV, in which the distal tip of the optic vesicle gives rise to the OCL, and then to the majority of the peripheral retina. Finally, we propose that there are distinct developmental histories, in terms of potential microenvironments and gene expressions, such that the future optic cup lip and thus peripheral retina is a different compartment than the central retina.

## Methods

### Embryo Handling and Harvest

Fertilized chicken eggs (Gallus gallus domesticus) were obtained from a local breeder (Petaluma Farms, CA) and incubated in a humid incubator at 39°C until Hamburger Hamilton stage (HH) 9/10. For in-ovo injections, embryos were visualized by opening the eggshell and injecting contrast ink below the embryo. Following injection, eggshells were resealed with parafilm (Pechiney Plastic Packaging, Chicago IL) and embryos re-incubated until ages required. For ex-vivo manipulations, embryos were cultured, ventral side up, according to the modified New Culture method [15]. Embryos were harvested by dissecting free of extra-embryonic tissues, fixation in 2% Paraformaldehyde in PBS for 2 hours at room-temperature, followed by extensive washing in PBS.

### Replication-incompetent Retrovirus

Proviral plasmids encoding GFP expression driven by a spleen necrosis virus [16], [17] were used for virus production as previously described [18]. Briefly, proviral and VSV-G plasmids were transfected into a Phoenix packaging cell line with stably incorporated gag and pol genes [19]. Virus supernatant was concentrated by ultracentrifugation and virus titer determined using a D17 cell line (Dog Osteosarcoma, ATCC catalog number CCL-183). Polybrene was added to a final concentration of 100 µg/ml to supernatant before storing aliquots at −80°C.

### Dye/Virus Injections

DiI (1,1′-dioctadecyl-3,3,3′3′-tetramethylindocarbocyanine perchlorate, Molecular Probes, Portland OR) and diO (3,3′-dioctadecyloxacarbocyanine perchlorate, Molecular Probes, Portland OR) were diluted in tetraglycol (T3396; Sigma Chemical Co.). Concentrated virus supernatant was mixed with CellTracker™ Green CMFDA (5-Chloromethylfluorescein Diacetate) (Molecular Probes) to permit confirmation of injection site. Dye or virus solution was back-loaded into pulled glass microcapillary pipettes. The vitelline membrane over the head was opened to expose the optic vesicle, capillary positioned, and dye/virus solution injected using a Harvard Instruments pressure injector to deliver the smallest observable amount to the basal surface of the optic vesicle epithelium. Images were captured to record labeling position and embryos re-incubated for between 36 and 72 hours.

### Imaging and Scoring

**Figure 1:**
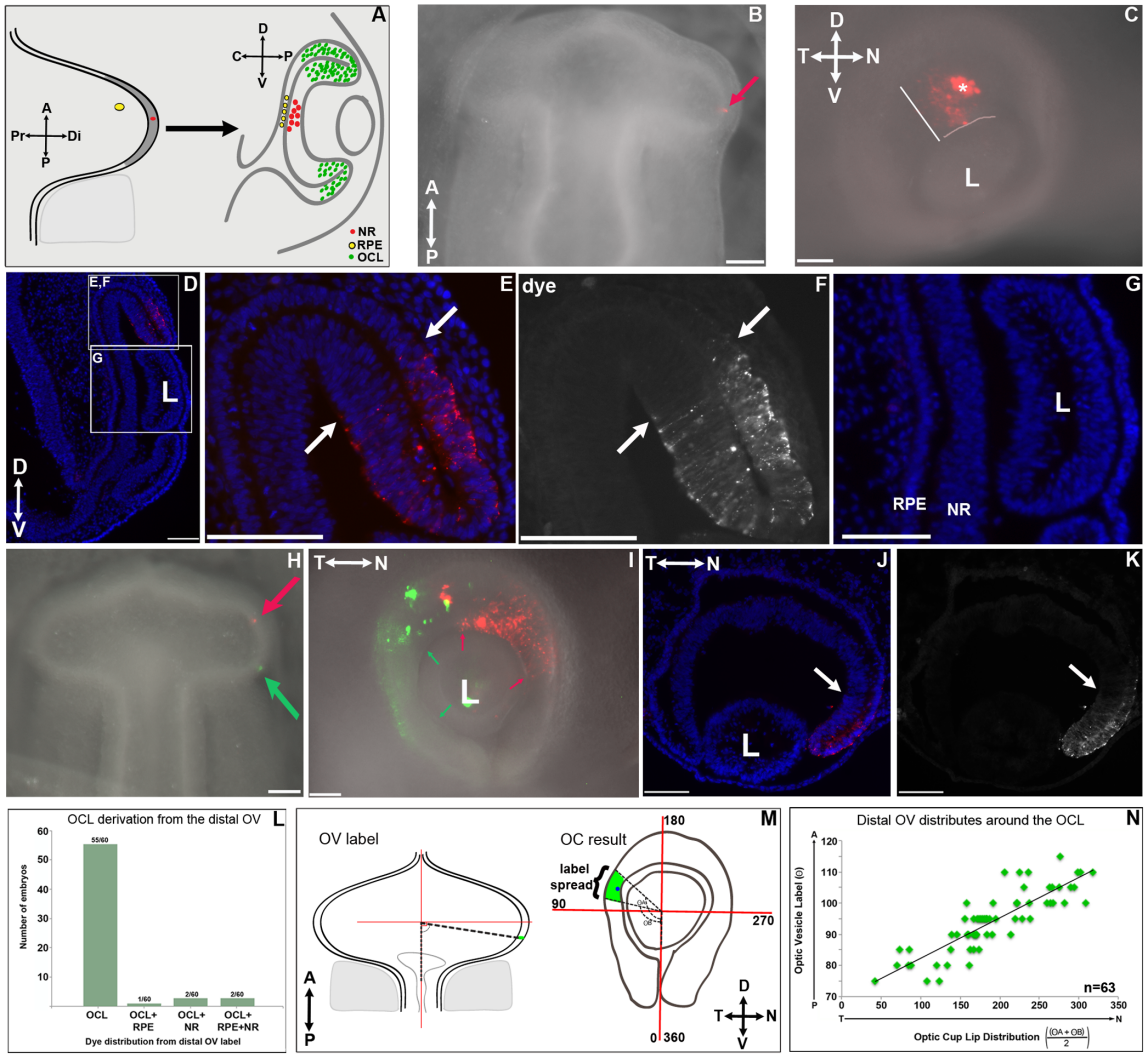
The Peripheral not Central Optic Cup Originates in the Distal Optic Vesicle. **A:** Scheme of the predicted distribution of dye directed to the OV of an HH10 embryo. The distal-most tip (red) is predicted to generate central neural retina. Central RPE is predicted to arise from the dorsal OV (yellow dots). OCL (green dots) origin is not known **B:** Dorsal view of a 12-somite embryo after DiI targeted to the distal OV (red arrow). **C:** Lateral view of the embryo in B following re-incubation. Dye distributed to the dorsal eye, from the OCL towards the central eye (white bar). Asterisk marks dye in the ectoderm. Pink line marks boundary between lens and OCL. **D–G:** Coronal sections of the embryo in B, C. **D:** Lower magnification for orientation. **E–F:** Dye in the dorsal OCL. Dye is present in the OCL and adjacent inner and outer layers (arrows). **G:** Dye is absent in both central neural retinal and RPE. **H:** DiI (red) and DiO (green) targeted to anterior and posterior distal OV. **I:** The embryo in H following re-incubation. DiO is distributed around the temporal OCL (green arrows) and DiI around the nasal OCL (red arrows). **J–K:** Transverse section through an embryo targeted at the anterior distal OV. Dye is restricted to the OCL and into the peripheral inner and outer layers (arrow). **L:** Graph showing dye distribution in the optic cup following distal OV labeling. Most embryos showed dye distributed to the OCL and excluded from the central neural retina or RPE. **M:** Scheme of the measuring strategy to analyze dye distribution. **N:** Plot of the distribution of distal OV targets against the midpoint of dye distribution around the OCL. Targeting through the posterior to anterior of the distal OV trends from temporal to nasal OCL. NR-neural retina, RPE-retinal pigmented epithelium, OCL-optic cup lip, L-lens, A-anterior, P-posterior, Pr-proximal, Di-distal, D-dorsal, V-ventral, T-temporal, N-nasal, OV-optic vesicle, OCL-optic cup lip. Scale bars = 100 µm.

Dye labeling was assessed in fixed embryos by capturing images with a Leica MZ16F stereomicroscope fitted with a Jenoptik ProgRes MF camera. Virus embryos were similarly imaged prior to fixation. Images were compiled using Adobe Photoshop v11.0.2 (Adobe Systems) and ImageJ v1.44o (National Institutes of Health, USA). Measurements of dye position were carried out using Image J. For distal OV label, the point where the embryonic midline crossed the longest axis of the optic vesicle was marked, a vector between the dye label in the distal OV and this point drawn, and the angle to the midline measured (Fig. 1M). For optic cups, a line was drawn to split the lens into equal nasal and temporal halves. The midpoint of the line was marked and the angles occurring at the intersection of this midpoint and the vector to the start/stop of dye in the OCL measured. These measurements translate the circumference of the OCL as 360 degrees, starting at the temporal choroid fissure and progressing clockwise to the nasal side of the choroid fissure. The measurements were plotted, OV position (y-axis) against the OC position (x-axis), to allow assessment of distribution of original OV labeling around the resulting optic cup.

### Cryo-sectioning and Immunohistochemistry

Embryo heads were sunk in PBS containing 20% sucrose and embedded in a 1∶1 solution 20% sucrose in PBS: OCT (Sakura Finetek, Torrance CA). Blocks were frozen on dry ice. Serial cryosections (10 µm) were collected and processed to visualize dye labeling or for immunohistochemistry. Sections for immunohistochemistry were blocked in PBS: 1% bovine serum albumen: 10% normal goat serum for 30 minutes before incubation in the same solution containing primary antibodies overnight at 4°C, followed by secondary antibody for 1 hour at room temperature. All sections were mounted in DAPI (4′,6-diamidino-2-phenylindole) containing mounting media (Vector Labs, Burlingame, CA). Primary antibodies used were mouse anti-Collagen IX clone 2C2 (Developmental Studies Hybridoma Bank [DSHB], Iowa City, IA), mouse anti-HuC (Molecular Probes, Portland, OR), rabbit anti-GFP (Rockland Immunochemicals, Gilbertsville PA), mouse anti-Lhx1 clone 4F2 (Developmental Studies Hybridoma Bank [DSHB], Iowa City, IA), rabbit anti-Lhx2/9 (a kind gift of Dr. Jane Dodd, Columbia University), diluted to 1∶250, 1∶100, 1∶2000, 1∶250, 1∶1000 respectively. Antibody labeling was visualized with complimentary secondary antibodies conjugated with Alexa488 or 594 (Molecular Probes, Portland OR). Comparable neighboring sections were similarly processed omitting primary antibody to control for non-specific secondary antibody labeling. Images were collected using a Nikon E800 microscope fitted with a Spot RT3 camera.

## Results

Prevalent models of eye development in higher vertebrates are based on the assumption that terminal eye compartments, that is the neural retina, RPE and the future ciliary body/iris, are largely fixed once the optic vesicle has morphed into a cup [20], [21]. There are few unbiased studies that address directly the optic vesicle (OV) origin of tissues within the optic cup (OC). We have recently described a substantial contribution from the OCL, at the margin of the OC, to the neural retina and retinal-pigmented epithelium. To determine the contribution of OV territories, we directly targeted the optic vesicle with vital dyes and assessed the subsequent dye distribution within the optic cup.

### The Optic Cup Lip, not Central Retina, Derives from the Distal OV

The distal OV is purported to give rise to neural retinal in the central region of the OC [22], [23]. To examine directly the contribution of the distal OV to the optic cup, focal dye injections were targeted around the distal optic vesicle and the embryos re-incubated for 36 hours, by which time the optic cup had formed. Embryos were examined in wholemount and after cryo-sectioning to score the distribution of dye. Optic cup labeling was scored according to the following criteria: *OCL label*, dye in the optic cup lip plus equal dye in anterior inner and outer layers; *Neural Retina label*, dye confined to the inner layer; *RPE label*, dye confined to the outer layer (Figure 1A). Models predict that dye targeted to the distal-most OV should contribute to the neural retina in the central optic cup (Fig. 1A, red dots). However, such labeling consistently resulted in dye in the dorsal optic cup, radiating from the OCL towards the central eye (Figures 1B, 1C, L). Sections through the plane of the dye showed that dye was present in the OCL and inner and outer layers of the peripheral optic cup (Figs. 1D–F). No dye was present in the inner or outer layers of the central aspect of the optic cup (Fig. 1D, G). This distribution is consistent with classification as OCL label.

Gene expression studies, which form the basis of a distal OV origin of neural retina, do not allow fine delineation of the distal extreme of the vesicle and neighboring territories around the edge of the OV. Therefore, dye was targeted around the distal OV, from where it abuts the mesoderm posteriorly to the corresponding point in the anterior OV, to examine differences in resulting dye distribution. If, as predicted, the distal OV is the origin of the neural retina, targeting around its extent should result in dye distribution into the central neural retina. In contrast, labeling any point of the distal OV resulted in dye only in the OCL (Fig. 1L 55/60 embryos). Other dye distribution patterns were infrequent: OCL+RPE (1/60 embryos), OCL+central neural retina (2/60 embryos), and OCL+ central RPE+central NR (2/60 embryos). Label directed at the anterior or posterior aspects of the distal OV distributed to the nasal or temporal OCL respectively (Fig. 1H–K). Again, dye was excluded from either the RPE or neural retina in the central optic cup.

These data demonstrate the distal optic vesicle gives rise solely to the OCL in the dorsal eye. To more stringently examine the distribution of dye from distal OV labeling around the circumference of the early optic cup, the position of dye label and resultant distribution was measured (Fig. 1M). Measuring the position of distal OV position relative to the midline and the resultant position of the dye in the OC (Fig. 1M) allowed direct evaluation of distribution of distal OV label to specific areas in the optic cup (Fig. IN). This mapping demonstrated that the entire distal optic vesicle contributed to OCL in a stereotypic pattern with posterior through anterior OV distributing from temporal through nasal OCL (Fig. 1N).

Together, these data identify that the distal optic vesicle does not give rise to neural retina, or RPE in the central optic cup but, instead, is the precursor of the optic cup lip for the dorsal eye.

### Derivation of Central Optic Cup Tissues

**Figure 2:**
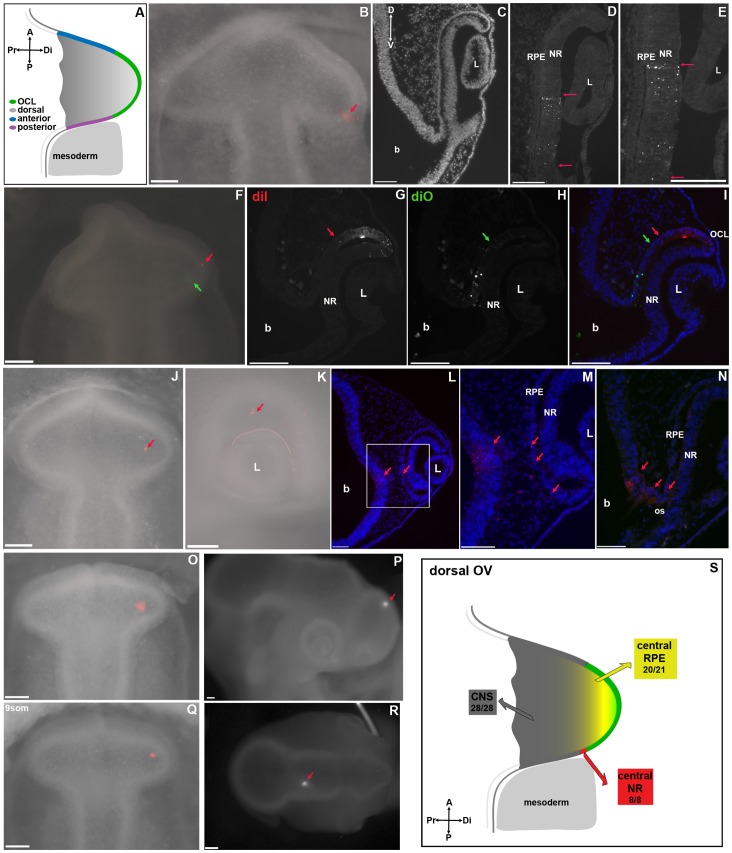
Identification of Central Optic Cup Fields in the Optic Vesicle. **A:** Schematic dorsal view of an optic vesicle at HH9/10 summarizing non-distal OV zones. **B:** Dye targeted to the posterior OV (arrow). **C–E:** Coronal sections of B following re-incubation. **C:** Low magnification. **D–E:** Higher magnification showing dye distributed in the central neural retina (red arrows). Dye is excluded from the OCL and posterior RPE. **F:** Dorsal view with DiI at the distal OV (red arrow) and DiO proximal (green arrows). **G–I:** Coronal section through the embryo in F following reincubation. **G:** DiI is distributed to the OCL. The central limit of DiI distribution is indicated (red arrow). **H:** DiO is distributed through the central RPE. The peripheral limit of DiO distribution is indicated (green arrow). **I:** Composite of G/H. DiI (red) in the OCL and DiO (green) in the central RPE do not overlap. **J:** Dorsal view of dye label proximal in the OV (red arrow). **K:** The same embryo following re-incubation. Dye is constrained towards the back of the eye (arrow). Pink line marks boundary between lens and OCL. **L–N:** Coronal sections through the embryo in J, K. **L:** DiI (red arrows) is in the central RPE and brain (red arrows) but absent from other eye domains. **M:** Higher magnification of the boxed area in L. DiI (red arrows) is distributed in the central RPE and in the brain. **N:** More temporally positioned section through the optic stalk. DiI is distributed from the central RPE through the optic stalk to the brain (red arrows). **O–R:** Optic vesicle labeling distributing in non-eye tissues. **O, Q:** Optic vesicle labeling in HH9 embryos. **P, R:** Distribution of dye in the CNS (arrows) following the labeling in O and Q respectively. No dye was distributed to the eye. **S**: Scheme summarizing dye distribution of OV dye labels into the central OC. Central RPE arose from the dorsal OV (yellow) and central NR from the posterior OV, adjacent to paraxial mesoderm (red). Proximal OV label distributed label to the CNS (grey). OV-optic vesicle, CNS-central nervous system, L-lens, NR-neural retina, RPE-retinal pigmented epithelium, A-anterior, P-posterior, Pr-proximal, Di-distal, D-dorsal, V-ventral, b-brain, L-lens. Scale bars = 100 µm.

The previous experiments demonstrate the origin of the OCL and also highlight that the central neural retina and RPE in the dorsal optic cup are not labeled by targeting the distal OV at HH9/10. To determine the origin of these tissues, the same dye labeling strategy was employed to expand beyond the distal OV. Injections were targeted to the dorsal OV, proximal to the distal edge, and anterior and posterior to the sites that gave rise to OCL (Fig. 2). Dye distribution was scored as previously, and designated neural retina or retinal pigmented epithelium when the label was confined to the respective single cup layer and not involving the OCL. Additionally, dye distribution was scored as CNS where dye was absent from the eye but present in the brain.

#### Central neural retina from the caudal OV

Neural Retina was the least frequent dye distribution observed. The posterior OV, immediately adjacent to paraxial mesoderm, was the only position that consistently gave rise to neural retina (Figs. 2B–E, S, red). The resulting neural retina label in the OC was only observed immediately dorsal to the optic stalk (Figs. 2D–E).

#### Central RPE from the dorsal OV

The origin of the RPE that overlies the central neural retina in the optic cup was examined. RPE-only dye distribution was observed from dye targeted to the dorsal OV, just adjacent to its distal extreme (Figs. 2F–I, S, yellow). As seen for central neural retina, discrete RPE label was situated dorsal to the optic stalk (Figs. 2F–H). Labeling the distal OV and adjacent dorsal OV demonstrated the linear relationship between the presumptive OCL and posterior RPE (Figs. 2F–I); distal OV label distributed to the OCL, including peripheral inner and outer layers (Figs. 2G, I), and proximal OV label was situated in a more central position within the optic cup (Figs. 2H, I).

In addition to dye distribution that was restricted to the central RPE, dorsal OV targeting also resulted in dye that distributed both to the central RPE and into the brain (Figs. 2 J–N). Following re-incubation, dye restricts to a discrete spot in the dorsal optic cup (Fig. 2K, arrow). Sections confirmed dye was in optic epithelium. In coronal section, dye was present in the central RPE and brain (Fig. 2L). In a nearby section, the label was seen in a continuous line of label through the central RPE, presumptive optic stalk, and adjacent brain (Fig. 2M), highlighting a gradation of eye and brain fate in the OV. Splitting embryos into two groups according to age, it was apparent that dye was distributed to the central RPE from the 50 µm of dorsal OV immediately proximal to its distal extreme in embryos containing 8–10 somite pairs. This RPE zone expanded to 100 µm in embryos containing 11–12 somite pairs (data not shown). In contrast the distance from the midline to the proximal limit of the RPE zone remained similar between the two ages suggesting a directed expansion of the RPE precursor territory from the distal OV, rather than a generalized growth of the entire tissue.

Targeting dye to areas outside regions shown to give OCL or central RPE and neural retina (Fig. 2A, grey shading) resulted in no dye label in the optic cup (Figs. 2O–R). The exact distribution of dye label into these territories beyond the OC was not fully examined here. Together with demarcation of the area of the OV that gives rise to eye, the data demonstrate that eye territory within the OV is restricted to the distal 50–100 µm of the OV between HH9 and 10.

As expected, the ventral optic cup was unlabeled after dorsal targeting at optic vesicle stages [11]. Although several studies involving surgical ablation have demonstrated that the ventral portion of the optic vesicle is required for eye morphogenesis, we did not find that the ventral most aspect gave rise to any label in the dorsal OC in unmanipulated embryos (data not shown) [5], [22], [24].

### The Relative Contribution of Central versus OCL Derived Precursors

**Figure 3:**
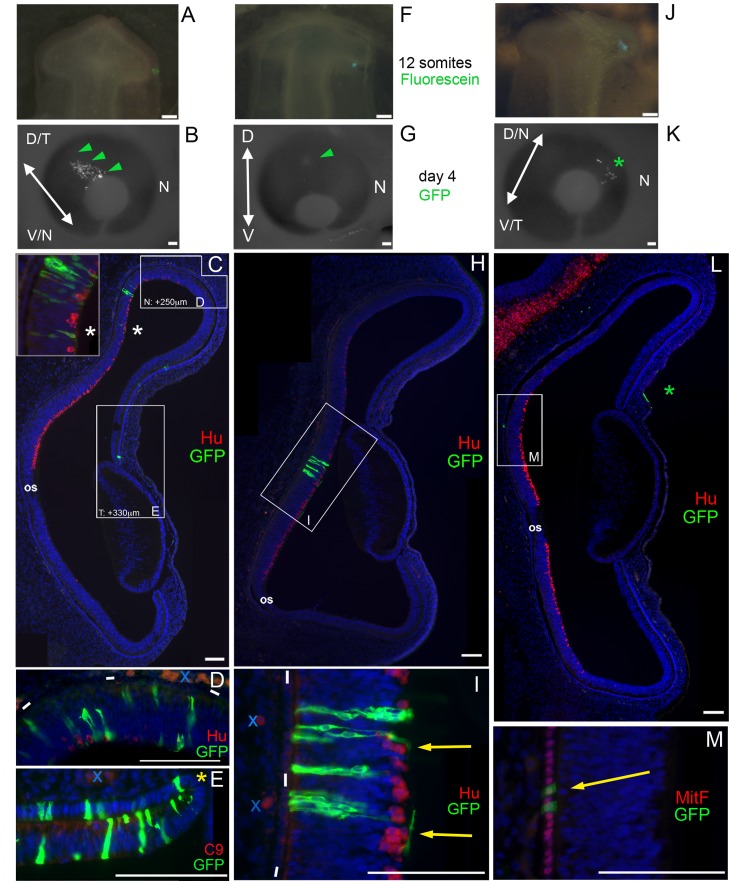
Central and Peripheral Optic Vesicle Domains are Traced to Differentiated Tissues of the Optic Cup. **A**: Dorsal view of HH10 embryo. GFP-expressing retrovirus, mixed with Cell-Tracker CMFDA, targeted to distal tip of optic vesicle (green fluorescein signal). **B:** Embryonic day 4; GFP+ cells visible in the eye (green arrowheads). Arrowed line indicates plane of sections. **C:** Section analysis of eye in B. Labeled cells organized in a spoke pattern extending from the optic cup lip (Box E) into the Hu+ neural retina (asterisk). **Inset panel:** Adjacent section of area at white asterisk, showing GFP-expressing neurons, including axon-extending ganglion cells. **D, E:** Adjacent sections of boxed zones in C; regions are slightly more nasal (N) or temporal (T) than boxed areas, indicated in µm. **D**: Pairs of labeled cells found at the edge of the Hu+ maturation zone. **E:** Labeled cells found in inner and outer layer at optic cup lip (yellow asterisk). **F:** Virus/CMFDA solution applied to the posterior optic vesicle. **G:** Day 4; indistinct GFP+ area visible through eye tissue (green arrowhead). Deep position of GFP within the inner layer accounts for weak signal. **H:** Section analysis of eye in G. Labeled cells found only in central neural retina. **I:** Magnification of boxed area in H; GFP+ neurons in retinal columns and extending axons towards optic nerve (yellow arrows). **J:** Virus/CMFDA applied to dorsal surface of optic vesicle, proximal to distal edge. **K:** Day 4; GFP+ cells visible in eye (asterisk). **L:** Sections reveal that visible GFP+ cells are in ectoderm over the optic tissue (green asterisk). Within eye, GFP+ cells found in central RPE (boxed area). **M**: Magnified view of boxed area on adjacent section, stained for MitF (RPE nuclei), showing GFP-expressing RPE cells (yellow arrow). Labeled cells found only in central RPE. 12 embryos were processed for virus evaluation. Antibodies as indicated, blue is DAPI-stained nuclei. Blue x’s indicate auto-fluorescent blood cells, white dashes on D,I indicate RPE layer. Scale bars = 100 µm D/T-dorsotemporal, V/N-ventronasal, D-dorsal, V-ventral, N-nasal, os-optic stalk.

Vital dye labeling allows tracking over shorter time periods, but dilution and spread make it unsuitable for longer-term fate tracking in proliferative cell populations. A GFP-expressing replication-incompetent retrovirus [18] was mixed with a fluorescent vital dye and targeted to territories that dye labeling had identified as precursors for central neural retina, central RPE, and OCL. The fluorescent tracker allowed confirmation of injection site and virus allowed tracking of fates through later stages of development, into tissues that express definitive fate markers (Fig. 3).

Dye labeling predicted that targeting virus to the distal OV would give rise to an OCL distribution in the optic cup. To effectively target only the distal tip, a small portion of surface ectoderm was removed and the dye-virus mixture applied externally to the OV. As predicted, wholemount examination of distal targets showed GFP expression in the peripheral eye at E4, extending from the OCL towards the central eye (Figs. 3A, B). Serial sections through the plane of the GFP expressing zone confirmed that GFP-expressing cells were found throughout the inner and outer layers of the optic cup. The labeled population extended through the Hu+ neural retina (Figs. 3C, inset and 3D) to the collagen IX-expressing optic cup lip (Fig. 3E). Virus labeling showed that, even after substantial growth, the cells originally at the distal tip of the optic vesicle do not populate the central retina but are widespread through more peripheral neural retina and anterior eye tissue.

In contrast, targeting virus to the posterior optic vesicle adjacent to the paraxial mesoderm gave deep labeling, removed from the peripheral eye, in wholemount views (Figs. 3F, G). Sections confirmed the presence of GFP-expressing cells confined to the inner, central neural retina layer (Figs. 3H,I), accounting for the weak signal seen in the wholemount embryo (Fig. 3G). GFP-expressing cells co-localized with Hu expression, confirming the neuronal identity of the tissue. Additionally, GFP expressing axons were present (Fig. 3I, yellow arrows). Matching the results with dye, this targeting distributed to central neural retina positioned dorsal to the optic stalk.

As with dye labeling, targeting the dorsal OV (Fig. 3J) distributed GFP-expressing cells to the central RPE (Fig. 3L,M). GFP-expressing cells were constrained to a small area dorsal to the optic stalk. Co-expression of MitF confirmed their identity as pigmented epithelium.

Viral-mediated labeling marked fewer cells than dye, but the permanent tagging allowed a clearer picture of the exact distribution of progeny of infected cells. Our previous studies have indicated a substantial contribution from the OCL to the OC [3], therefore we were interested in the overall extent of label after targeting the precursor of the OCL versus the central NR or central RPE.

Analysis of the GFP+ content of every section of the infected eyes showed that the OCL-labeled populations extended over 1800 µm from peripheral to central. The section shown in Fig. 3C captured both the most peripheral (at the OCL) and most central positions. In comparison, after labeling the presumptive central neural retina in the OV, GFP+ cells were found in 18 adjacent sections, in the same relative position within the optic cup; Fig. 3H represents a section from the center of the area. After targeting the RPE domain, the GFP+ population was spread over approximately 22 sections; one representative 10 µm section from the middle of the population is presented (Fig. 3L). Again, GFP+ cells were in the same relative position within the central aspect of the optic cup.

The OCL-labeled populations were followed over 80 adjacent serial sections; that is, there were GFP+ representatives in every one of the 80 sections. The boxed areas in Fig. 3C show the relative position of portions of the optic cup that are filled with GFP+ cells in adjacent sections slightly nasal (Fig. 3D) or temporal (Fig. 3E); the section plane did not allow visualization of the complete central-peripheral spread of GFP-expressing cells in a single section. These data confirm and extend our previous demonstration that OCL-derived cells populate the neural retina during embryonic development [3].

### Sub-domains within the HH10 Optic Vesicle

**Figure 4:**
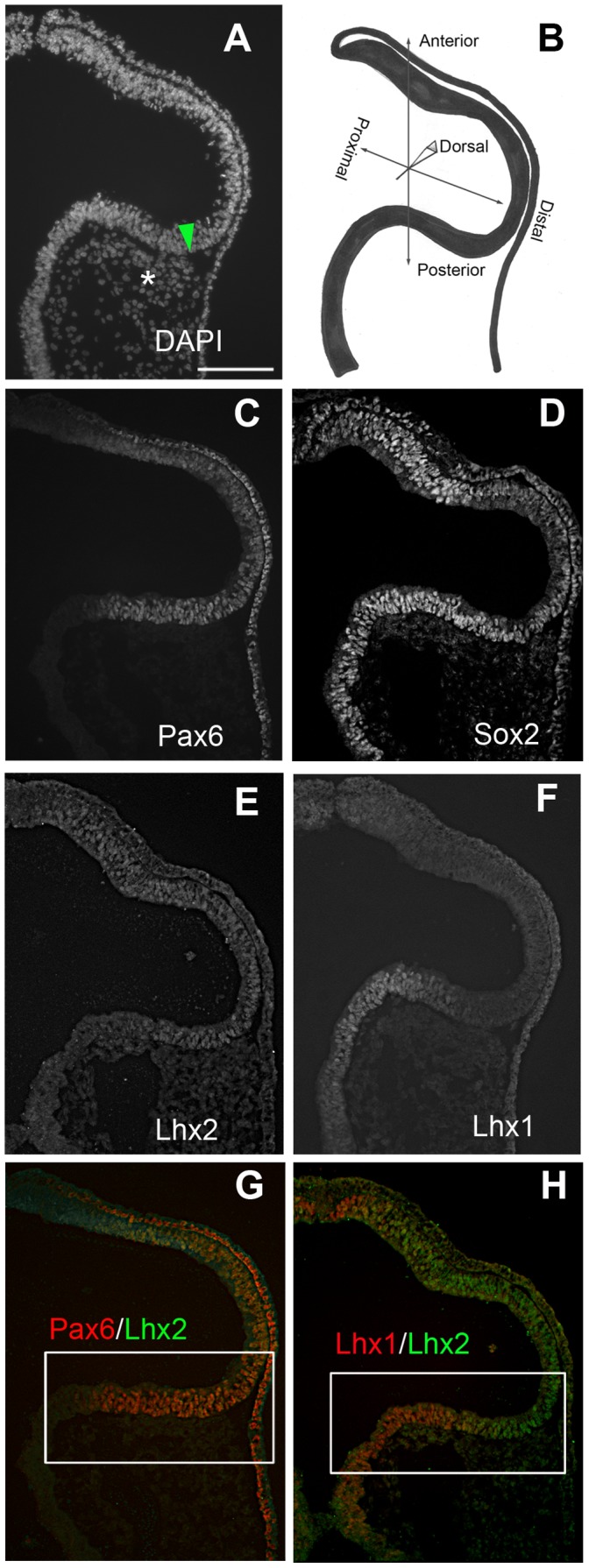
The Expression of Eye Field Transcription Factors subdivides the Optic Vesicle. **A–H**: Coronal sections through HH10 optic vesicles immuno-labeled as indicated on panels. **A:** DAPI stained coronal section, to highlight the relevant tissues. Asterisk indicates the paraxial mesenchyme in contact with the posterior optic vesicle. Green arrow indicates the targeted site of injection for labeling the central neural retina in previous figures. **B:** Graphic representation of sections with axes labeled for orientation. **C–F:** Single immunostaining as indicated. **G, H:** Double immunostaining as indicated. Scale Bar in A applies to panels C–H. Scale bars = 100 µm.

The expression patterns of several relevant eye transcription factors were examined in coronal sections through HH10 optic vesicles, to highlight the site of injection that labels central neural retina versus OCL. (Fig. 4). We looked at the expression of three eye field transcription factors that have been shown to induce ectopic neural retina tissue: Sox2, Pax6 and Lhx1 [25], [26], [27]. We also looked at Lhx2, a LIM-domain family member related to Lhx1; LIM family members identify regional sub-patterning in several neural regions [28], [29], [30]. When viewed in a coronal section, Sox2 Pax6 and Lhx2 are found throughout the proximal-distal axis of the OV, in a basically overlapping pattern. (Fig. 4C–E). Lhx1, in contrast, was expressed in the posterior portion of the OV and into the neural tube (Fig. 4F). Lhx1 overlapped with Lhx2 (and Pax6) in the posterior OV, where the paraxial mesoderm contacts the neuroepithelium (Fig. 4G, H). Thus, at HH12, the future central neural retina can be defined as a sub-domain that expresses Pax6, Lhx2, Sox2 and Lhx1. In comparison, the future OCL domain does not express Lhx1.

The data here tested a prevalent model of eye development, whereby the distal optic vesicle gives rise to the central portion of the neural retina. We found instead that the distal OV is the precursor of the optic cup lip, the most anterior part of the eye. The concept that OC tissues are segregated in the OV was also examined. We found that, for the dorsal eye, three optic cup domains could be demarked in the vesicle: central neural retina, central RPE, and OCL.

## Discussion

Previous optic vesicle mapping studies placed the central neural retina at the distal OV [22], [23]. Upon invagination this domain is thought to displace to the back of the eye, becoming the central retina [31]. Our previous studies identified the OCL as a progenitor pool for more peripheral regions of the OC: peripheral retina, ciliary body, and iris. To examine the relationship between these central and peripheral eye domains at earlier developmental stages, we undertook to fate map the OV to determine 1) the origin of the OCL and 2) if the central/peripheral retinal domains represent distinct units in the eye primordia.

**Figure 5:**
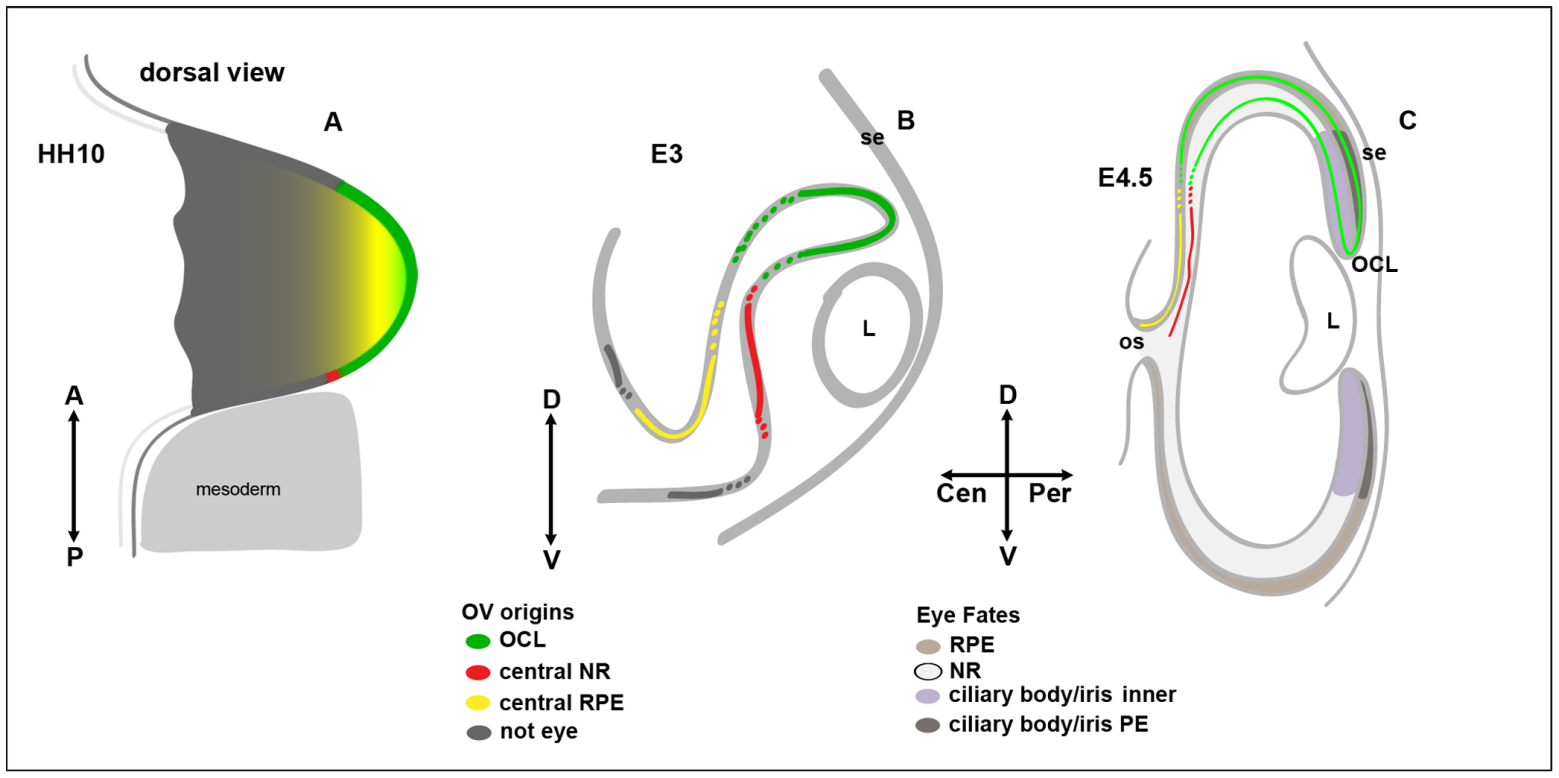
Mapping Central and Peripheral Eye Domains. Schematic summary of optic vesicle fate mapping results. **A:** Three distinct optic cup domains can be defined in the dorsal optic vesicle at HH9/10; OCL at the distal OV (green), central RPE situated more proximally (yellow), and central NR at the posterior OV (red). **B:** Distribution of dye from OV labeling in a forming optic cup at E3. Distinct central NR (red) and RPE (yellow) zones are constrained to the optic cup dorsal to the optic stalk. Distal OV/OCL label (green) distributes equally to inner and outer layers at initial OC stages. **C**: Representation of labeling results correlated with fate domains of NR, RPE, and anterior inner and outer eye fates at E4.5. Central RPE and NR zones remain restricted to the central eye. An anterior bias is evident in the extent of OCL derived OC tissue, which traverses the boundary between NR and presumptive ciliary body/iris. A-anterior, P-posterior, D-dorsal, V-ventral, Cen-central, Per-peripheral, L-lens, RPE-retinal pigmented epithelium, NR-neural retina, OV-optic vesicle, OCL-optic cup lip, OC-optic cup, se-surface ectoderm, os-optic stalk.

Compared to previous lineage analyses, we recorded three significant differences in OV topography (Fig. 5):

1. The central neural retina can be traced from a distinct domain at the posterior aspect of the OV;
2. The future growth center at the OCL can be traced from the distal tip of the OV, and directly contributes extensively to the peripheral neural retina during embryonic stages;
3. The central RPE can be traced from the dorsal OV.

These data highlight dual origins of the neural retina from OV stages, and marker expression suggests a similar compartmentalization (Fig. 4). Also, an ongoing peripheral growth bias during optic cup morphogenesis is uncovered that reflects well documented central to peripheral differentiation gradients that occur concurrent with eye development. Superficially this observation would appear to concur with current models depicting an early segregation of eye fates. However, permanent tagging of OV territories with retrovirus demonstrates that the OV domains reflect the graded central- peripheral maturation of the optic cup and that, in contrast to the models, the OCL territory does not predict a terminal OC fate as ciliary body/iris (Fig. 5).

### Embryonic Basis for a Peripheral versus Central Subdivision in the Adult Retina

The central eye domains that emerge in the OV are positioned in distinct territories. This study does not address whether these tissues are already determined towards a single fate at the time of labeling. However, it is apparent that their positioning will expose them to specific micro-environments likely to influence their final fate. The future central neural retina of the optic vesicle, at the same ages used for tagging experiments, co-expresses Pax6, Lhx1, Lhx2, and Sox2; Lhx1 expression is highest in that posterior quadrant and the domain of expression does not enlarge at later stages (data not shown, [27]). These four factors are part of a transcriptional network that can induce ectopic eyes outside of the nervous system in Xenopus [32]. Pax6, Sox2 and Lhx1 each individually can induce neural retina tissue from the RPE in the chick when mis-expressed [25], [26], [27]. Lhx2 cannot induce ectopic eye tissue by itself, and was not added to the cocktail in the Xenopus model, but is apparently involved, as it always induced during ectopic eye formation [27], [32]. The ultimate mechanism through which these factors induce/integrate to create neural retinal fields is still unknown. Pax6 and Lhx2 are required for the transition from OV to OC, and they are independent, in that they do not regulate the others expression. [33], [34], [35]. Mechanistically, Pax6 is proposed to keep the OV in an undifferentiated state, and Lhx2 may work to maintain BMP expression at OV stages [33], [34], [36]. Specific mechanisms for the role Sox2 and Lhx1 in optic tissue are still unknown, although both Sox2 and Lhx1 upregulate FGF; it is not clear if this is direct or indirect [26], [27]. It is premature to assume that the melange of nuclear localized protein expression in posterior versus distal optic vesicle is tantamount to specifying the central neural retina. Rather, it is an indication that there is some sub-division in this stage OV.

At HH12+ (17 somites), labeling applied using the same targeting criteria (the junction of the posterior optic vesicle and the contacting mesoderm) labels the temporal OCL; the central neural retina domain has shifted, as expected, towards the surface ectoderm (data not shown). A role for sheet and/or cell movement during the transition from vesicle to cup is possible. Recently, experiments comparing fish and chick eye development highlighted rotational cell movements during OV growth in the fish, and it was proposed that similar movements occur during chick OV development [37]. This could be one explanation, and the easiest. However, none of our labeled embryos produced patterns consistent with a rotational pattern of growth. In cases where embryos were taken out at early stages of OC initiation, or in our tracing of the linear expansion of the RPE domain on the dorsal OV, we found more support for the model of linear proximo-distal growth from the neural tube/diencephalon. [38], [39]. Superimposing these movements directly onto the chick embryo might be premature; the fish optic vesicle has unique developmental features not found in terrestrial vertebrates. For instance, in fish the OV begins as a solid ball of cells, not as an epithelial sheet that extends from the neural tube [40]. Also OC formation is dependent on cell proliferation in mouse, chick and frog, but not so in fish [37], [41], [42], [43], [44]. Our continuing studies on the specification and development of the central neural retina will address how cell migration and/or proliferation integrate to position this compartment within the OC.

Lineage analysis by vital dye labeling was used by Dutting and Thanos [22] and their study produced the textbook model of OV patterning, in which the central neural retina is established at the distal tip of the OV. Generally, lineage studies have excluded the peripheral retina, the RPE, and ciliary body/iris from analysis, and older endpoints were assayed, which accounts for why an OCL-driven growth pattern has never been described [11], [22], [23], [45], [46]. Also, because lipophilic dye injections can leave crystals within target tissues, it is relevant to distinguish between primary and potential secondary labelings from the included crystal. In this study, DiI/DiO label was scored only if it was clearly associated with a cell membrane; eyes with large ultra-bright crystals were not included (Fig. 1). In our methods, fluorescent dye was added at minimal enough levels that it had diluted beyond detection after 36 hours; this is in the same range of time that electroporated transcripts such as GFP dilute out of chick cells and therefore it is not to be expected that DiI labeled cells should be observable much past 2 days after application in a proliferative tissue [47]. Finally, permanent labeling via retrovirus replicated the dye findings and revealed the final position of labeled cells within a clearly formed and compartmentalized optic cup (Fig. 3).

We have concentrated in this study on the dorsal optic cup. Ventral OV labeling was carried out to examine any contribution of the ventral OV to the dorsal OC. Labeling the ventral OV, at the stages used here, distributed to the optic stalk/presumptive choroid fissure and brain but never to the dorsal OC (data not shown). Our preliminary observations indicate that the ventral OC is formed later than the dorsal, from cells that occupy the choroid fissure, and which originated in the ventral OV.

Whether our fate map of the avian eye is directly transferable to understanding eye growth in the mammalian eye remains open to testing. The direct lineage analysis presented here is not easily done in the mammalian system. However, in support of conserved mechanisms, cre-driven lineage analysis in the eye has been interpreted as presenting distinct origins for peripheral and central retina [48].

### Sustained Output from the Optic Cup Lip Correlates with the Central-to-peripheral Axis

We have uncovered distinct OV origins of central and peripheral eye tissues, revising earlier models of eye morphogenesis (Fig. 5). We propose that only a limited central portion of the retina is distinct in the optic vesicle. The central neural retina domain was uncovered in the posterior OV adjacent to the paraxial mesoderm (green arrowhead in Fig. 4A). Sections through newly formed optic cups showed that after morphogenesis, this domain is situated directly behind the lens. Likewise, the central RPE, labeled on the dorsal surface of the optic vesicle, comes to rest in an equivalent position in the outer layer of the OC.

The peripheral eye originates at the distal OV. Dye labeling shows that the total of the labeled spots comprise a field of cells that form into the OCL around the dorsal eye. As with direct OCL targeting [3], labeling OCL precursors results in sustained distribution of progeny to both inner and outer OC layers. The same distal OV labeling protocol with a GFP-expressing replication-incompetent retrovirus, to extend the observable period, demonstrates that the cells of the distal tip create a population that extends from the OCL through the peripheral retina but do not extend into the central neural retina (Fig. 4). This population inhabits a zone that expresses neuronal differentiation markers, and crosses into a previously postulated position-dependent expansive “pre-neurogenic zone”, that is expected to mature into a neurogenic zone as the microenvironment changes [49].

While timing of labeling almost certainly allows distinct central OC precursor to be identified, more importantly, it highlights differences in embryonic history that may contribute to overt functional differences between central and peripheral retina: central retina emerges early from a common eye niche and is never incorporated into the OCL niche that distributes progeny throughout the remainder of the eye. Further study is required to understand both external patterning that acts upon central and peripheral eye domains leading to fate determination, and the intrinsic cell changes that may reflect timing of emergence from a common progenitor niche. The distinct micro-environments that central RPE and neural retina precursors occupy in the optic vesicle allow speculation of adjacent tissues that may be involved in fate assignment and differences in protein expression will likely allow perturbation of these compartments. Similarly, examination of differences in cell potential along the proximo-distal axis of the optic vesicle may unveil stepwise commitment of cells towards a particular eye fate. With the resolution afforded by the finite unbiased fate tracing herein, stringent interpretation of optic vesicle perturbation is possible. The demarcation of two distinct retinal domains, that are established during embryogenesis, will allow renewed evaluation of human diseases that are specific to either retinal sub-domain.
